# Bending Resistance Covalent Organic Framework Superlattice: “Nano-Hourglass”-Induced Charge Accumulation for Flexible In-Plane Micro-Supercapacitors

**DOI:** 10.1007/s40820-022-00997-0

**Published:** 2022-12-30

**Authors:** Xiaoyang Xu, Zhenni Zhang, Rui Xiong, Guandan Lu, Jia Zhang, Wang Ning, Shuozhen Hu, Qingliang Feng, Shanlin Qiao

**Affiliations:** 1 College of Chemistry and Pharmaceutical Engineering, Hebei University of Science and Technology, Shijiazhuang, 050018 People’s Republic of China; 2 State Key Laboratory of Chemical Engineering, East China University of Science and Technology, Shanghai, 200237 People’s Republic of China; 3 School of Chemistry and Chemical Engineering, Northwestern Polytechnical University, Xi’an, 710072 People’s Republic of China; 4 Hebei Electronic Organic Chemicals Engineering Center, Shijiazhuang, 050018 People’s Republic of China

**Keywords:** In-plane micro-supercapacitor, Covalent organic framework, Free-standing nanofilm, Superlattice, Bending resistance

## Abstract

Covalent organic framework (COF) film with highly exposed active sites is considered as the promising flexible self-supported electrode for in-plane micro-supercapacitor (MSC). Superlattice configuration assembled alternately by different nanofilms based on *van der Waals* force can integrate the advantages of each isolated layer to exhibit unexpected performances as MSC film electrodes, which may be a novel option to ensure energy output. Herein, a mesoporous free-standing A-COF nanofilm (pore size is 3.9 nm, averaged thickness is 4.1 nm) with imine bond linkage and a microporous B-COF nanofilm (pore size is 1.5 nm, averaged thickness is 9.3 nm) with *β*-keto-enamine-linkages are prepared, and for the first time, we assembly the two lattice matching films into sandwich-type superlattices via layer-by-layer transfer, in which ABA–COF superlattice stacking into a “nano-hourglass” steric configuration that can accelerate the dynamic charge transportation/accumulation and promote the sufficient redox reactions to energy storage. The fabricated flexible MSC–ABA–COF exhibits the highest intrinsic *C*_V_ of 927.9 F cm^−3^ at 10 mV s^−1^ than reported two-dimensional alloy, graphite-like carbon and undoped COF-based MSC devices so far, and shows a bending-resistant energy density of 63.2 mWh cm^−3^ even after high-angle and repeat arbitrary bending from 0 to 180°. This work provides a feasible way to meet the demand for future miniaturization and wearable electronics.

**Supplementary Information:**

The online version contains supplementary material available at 10.1007/s40820-022-00997-0.

## Highlights

- Covalent organic framework (COF) superlattices were assembled by free-standing COF nanofilms based on the *van der Waals* force for the first time.
- Geometry-induced “nano-hourglass” steric configuration in COF superlattice can provide rapid charge transfer/accumulation at heterojunction interface for in-Plane Micro-supercapacitors.
- The prepared MSC exhibited a high energy density of 63.2 mWh cm^−3^ even after high-angle and repeat arbitrary bending from 0 to 180°.

**Supplementary Information:**

The online version contains supplementary material available at 10.1007/s40820-022-00997-0.

## Graphical abstract

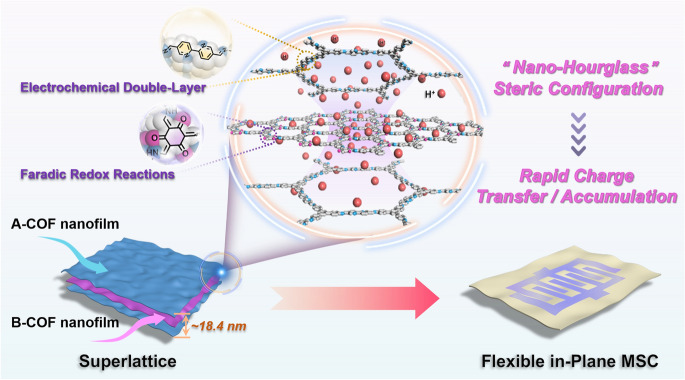

## Introduction

The extensive application of wearable/flexible electronics in implantable medical devices, intelligent electronic skins and foldable displays, etc., is driving strong demand for in-plane micro-supercapacitor (MSC) devices [1–3]. The unremitting efforts on in-plane MSC focus on the large-size and flexible film electrodes, contributing to the high dynamics transmission and energy density even in arbitrary bending [4–7]. Based on electrochemical double-layer capacitance and pseudocapacitance two energy storage mechanisms, supercapacitors can be classified into electrochemical double-layer capacitors and pseudocapacitors [8]. Electrode active materials typically increase specific surface area, conductivity and modify redox active groups by combining two energy storage mechanisms to improve the comprehensive performance of the device [9].

Covalent organic framework (COF) films with high structural integrity and internal continuity were successfully prepared and used to fabricate MSC interdigital electrodes in our previous work, space-partitioning and metal coordination two strategies were used to in-situ modify the COF film on the basis of energy storage mechanisms, exhibiting unexpectedly high capacitive performance and energy density [10]. Although the capacitance is greatly enhanced by ionic liquid modification and metal doping, the effective synergistic utilization of the intrinsic pores and redox-active groups to enhance both the electrochemical double-layer capacitance and pseudocapacitance at the same time remains a challengeable work for MSC device.

Superlattice configuration with the alternating assembly of different two-dimensional (2D) materials have aroused strong interest. Recently, superlattice heterostructures of organic/inorganic materials have been applied to charge storage and showed tremendous potential [11–13]. The unique 2D heterogeneous structure significantly affects the charge distribution/modulation at the interface, and exploiting the synergistic effect of two intimate contacted laminations to enhance the supercapacitor performances, not the simple combination of two individual components [14–18]. Tight and homogeneous interfacial bonding is the prerequisite for excellent charge storage performance [19–22]. However, most superlattices assemblies are performed by exfoliation, dispersion or stirring, which inevitably leads to structural impairment at the molecular-level. It is still a challenge to obtain large-size, intact and ordered superlattices with nano-thickness [23]. The COF films prepared by surfactant monolayer-assisted interfacial synthesis (SMAIS) strategy exhibit large size, smoothness and ease of transfer [24], which maybe provide a possible way to obtain periodically complete and ordered 2D COF superlattices with high quality and controlled stacking. In addition, each COF layer can be pre-designed with active units and pore size based on the energy storage mechanisms, acting as both host and interface materials to effectively promote charge transfer/storage and further improve electrochemical performance [25, 26]. This means that 2D COF superlattices can realize the control of thickness and structure concurrently, and utilize the properties of each isolated layer to derive synergistic effects, which may be an effective way to obtain satisfactory overall supercapacitor performance [26].

Herein, a mesoporous free-standing COF film (defined as A-COF) with imine bond links and a microporous COF film (defined as B-COF) with *β*-keto-enamine-linkages were prepared by the SMAIS method at atmospheric pressure and room temperature, and for the first time, we using the two films to assemble superlattices self-supported electrode based on electrochemical double-layer capacitance and pseudocapacitance two energy storage mechanisms. The designed concepts are as follows: (i) the COF structures linked by covalent bonds allow them to bend and stack without being broken, and stacking them in long-range periodic to obtain uniform nano-thickness superlattice, which provides good ion accessibility to the inner surface of the active site [27, 28]; (ii) superlattices enables the free assembly of the COF films, exploiting the synergistic effect of each layer to further improve the electrochemical properties. The final performance of the fabricated interdigital MSC device verified an amazing discovery that the superlattice stacking into a “nano-hourglass” ABA steric configuration, can form a geometry-induced rapid charge transfer/accumulation at heterojunction interface, and synergistic effect in electrochemical double-layer and faradic redox reaction to promote the energy storage. As will be described below, the superlattice showed remarkably high activity as flexible electrodes for in-plane MSC.

## Experimental Section

### Materials

*N,N*-dimethylformamide (DMF), *t*-butoxybis(dimethylamino)methane, sodium dodecylbenzene sulfonate (SDBS), 1,3,5-tris(4-aminophenyl) benzene (TAPB), 2,4,6-trihydroxybenzene-1,3,5-tricarbaldehyde (TP), ether, ethanol and acetone were purchased from Damao Chemical Reagent Factory (Tianjin, China). 5,5′-Dimethyl-2,2′-bipyridine was obtained from Shanghai Bide Medical Technology Co., Ltd. The CH_2_Cl_2_, THF, sodium periodate, EtOAc, MgSO_4_, acetic acid, poly (vinyl alcohol) PVA, H_3_PO_4_ and hydrochloric acid chemicals were obtained from Aladdin Industrial Corporation (Shanghai, China). All chemicals were used without further purification.

### Synthesis of 2,2'-Bipyridine-5,5'-dicarbaldehyde (Bpy)

Bpy was synthesized according to the procedures described in the studies [29]. Firstly, a mixture of 5,5'-dimethyl-2,2'-bipyridine (100 mg, 0.54 mmol), DMF (1 mL), and *t*-butoxybis(dimethylamino)methane (1 mL) was added into a Pyrex tube. The solution was subjected to three cycles of freeze–pump–thaw treatment. Then the reaction was allowed to proceed at 120 °C for 5 days in an oven. After which time, a yellow powder was collected by filtration, washed with ether (3 × 2 mL), and dried under vacuum at 40 °C. 1H NMR (CDCl_3_, 400 MHz):* δ* (ppm) 8.41 (s, 2H), 8.13 (d, 2H), 7.56 (s, 2H), 6.87 (d, 2H), 5.21 (s, 2H), 2.86 (s, 12H).

Secondly, 5,5'-bis(2-dimethylaminovinyl)-2,2'-bipyridine (100 mg, 0.34 mmol) was dissolved in a mixture of CH_2_Cl_2_ (4.5 mL) and THF (15 mL). The solution was added an aqueous solution of sodium periodate (0.553 g, 2.6 mmol) in 3 mL water. Then the mixture was stirred at room temperature for 20 h. The solid was filtered off and the solution was concentrated and extracted with EtOAc. The organic phase was dried over MgSO_4_ and evaporated to afford a crude product. 1H NMR (DMSO, 400 MHz): δ (ppm) 10.20 (s, 2H), 9.23 (s, 2H), 8.65 (d, 2H), 8.43 (t, 2H).

### Synthesis Method of A- and B-COF Films

The deionized water (100 mL) was injected into a crystal dish (*d* = 120 mm, *h* = 60 mm) to form a static air–water interface. Then SDBS (20 μL, 1 mg mL^−1^ in chloroform solvent) was dispersed on the interface. After solvent evaporation (~ 30 min), the monomer TAPB (351 μL, 1 mg mL^−1^ in 0.12 M HCl solution) was injected and dispersed into water for 1 h. Afterwards, the monomer Bpy (318 μL, 1 mg mL^−1^ in 0.12 M HCl solution) was added to the aqueous phase. The reaction was maintained at room temperature by employing acetic acid (0.01 M, 2 mL) as a catalyst. After 1 week, the A-COF film was obtained. Similarly, B-COF film was prepared in the same procedure, except that the two monomers involved were TAPB (351 μL, 1 mg mL^−1^ in 0.12 M HCl solution) and TP (210 μL, 1 mg mL^−1^ in 0.12 M HCl solution), respectively.

### Non-electrochemical Characterization

The morphology of prepared films was observed by optical microscopy (OM, Leica ICC50 W), atomic force microscopy (AFM, Bruker Dimension Icon), scanning (SEM, JEOL) and transmission electron microscopy (TEM, JEOL/JEM-2100). Solid ^13^C NMR experiments were characterized using a Bruker 400 MHz. Fourier transform infrared spectrum (FT-IR, Thermo Scientific Nicolet iS10 spectrometer), Raman spectra (Horiba JY, LabRAM ARAMIS) and X-ray photoelectron spectroscopy (XPS, Thermo Scientific K-Alpha spectrometer).

### Electrochemical Characterization

The electrochemical measurements including cyclic voltammetry (CV), galvanostatic charge–discharge (GCD) and electrochemical impedance spectroscopy (EIS) in the three-electrode system containing the prepared working electrode, platinum mesh counter electrode, and AgCl/Ag reference electrode were carried out in 1 M H_3_PO_4_ aqueous solution on a CHI660E electrochemical workstation. The working electrode was a glass carbon electrode (5 mm in diameter) coated with two COF films or four superlattices samples. EIS measurement was conducted at the open circuit potential with an amplitude of 5 mV in a frequency range from 0.01 Hz to 100 kHz.

The specific areal capacitances (*C*_S_, mF cm^−2^) of two COF films and four superlattices samples in the three-electrode system on the basis of GCD curves can be obtained according to the following Eq. (1):

1 $$ C_{S} = \frac{J \times \Delta t}{{\Delta V}} $$

where *J* is the current density (mA cm^−2^) of charge/discharge, *Δt* is the discharged time (s), *ΔV* is voltage output window (V).

The contribution ratio of surface capacitance (${k}_{1}v$) and diffusion-controlled (${k}_{2}{v}^{1/2}$) were calculated for four COF superlattices by Eq. (2) [30]:

2 $$ i = k_{1} v + k_{2} v^{1/2} $$

The properties of MSC were investigated by CV technique in a two-electrode system. The electrochemical measurements of MSC devices were carried out by using the VersaSTAT 3 electrochemical workstation. The MSC was assembled by using the prepared interdigital electrode and PVA/H_3_PO_4_ gel electrolyte. The interdigital electrodes of in-plane MSC devices were prepared by mask-assisted method (Fig. S1), transferring superlattices to PTFE substrate via the slowly discharge reaction solvent. The PVA/H_3_PO_4_ gel electrolyte was prepared by mixing 6 g H_3_PO_4_ and 6 g PVA in 60 mL deionized water and heated up to 90 °C for 1 h under vigorous stirring.

The specific areal (*C*_A_, mF cm^−2^) and volume (*C*_V_, F cm^−3^) capacitances of MSC were calculated according to the following Eqs. (3) and (4) [31]:

3 $$ C_{A} = \frac{1}{{2v \times A \times \left( {V_{f} - V_{i} } \right)}}\mathop \int \limits_{{V_{i} }}^{{V_{f} }} I\left( V \right)dV $$

4 $$ C_{V} = \frac{1}{{2v \times V \times \left( {V_{f} - V_{i} } \right)}}\mathop \int \limits_{{V_{i} }}^{{V_{f} }} I\left( V \right)dV $$

where *ν* is the scan rate (V s^−1^), ${V}_{f}$ and ${V}_{i}$ are the integration voltage limits of CV curve, $I(V)$ is the voltametric current (A), *A* and *V* are the area (cm^2^) and volume (cm^3^) of the entire MSC device.

The volume energy density (*E*, Wh cm^−3^) and power density (*P*, W cm^−3^) of MSCs can be further calculated by Eqs. (5) and (6) [31]:

5 $$ E = \frac{1}{2} \times C_{V} \times \frac{{\left( {\Delta V} \right)^{2} }}{3600} $$

6 $$ P = \frac{E}{\Delta t} \times 3600 $$

where $\Delta V$ is the discharge voltage range (V) and $\Delta t$ is the discharge time (s).

### Density Functional Theory (DFT) Calculations

The electrostatic potential distribution map was obtained by the DMol3 calculation with Materials Studio software. During geometry and frequency optimizations, the calculations were carried out with the GGA functional and the DND basis set. The cut-off energy was set to 2 × 10^–5^ Ha for the calculation.

## Results and Discussion

### Superlattices Assembly and Sample Characterizations

**Scheme 1:**
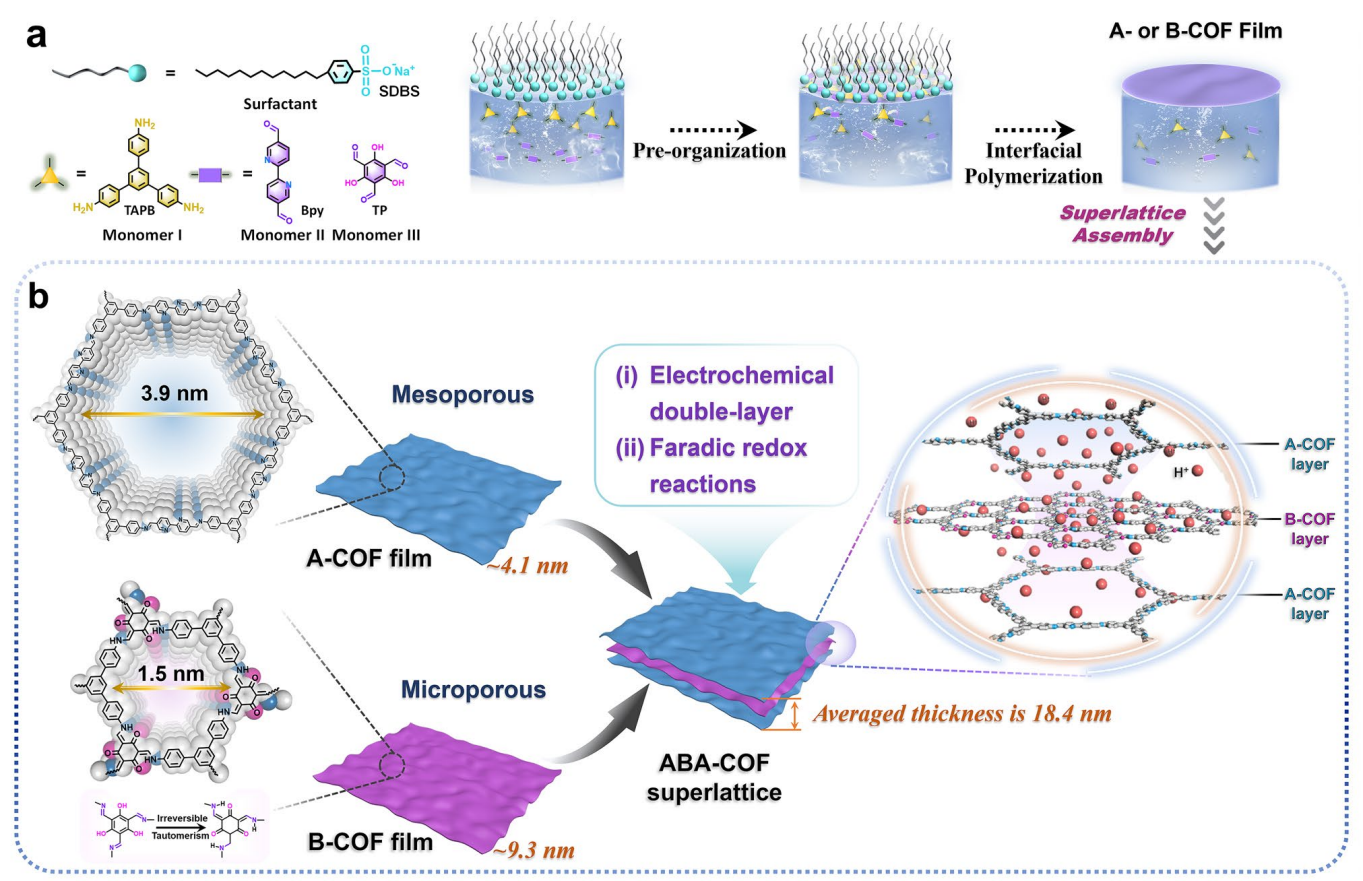
**a** Schematic synthesis of A- and B-COF films using the SMAIS method. **b** Illustration of assembling A- and B-COF films to ABA-COF superlattice

The superlattices were assembled with A- and B-COF nanofilms by *van der Waals* force using layer-by-layer transfer. As shown in Scheme 1a, monomer I (TAPB) and monomer II (Bpy) are combined to synthesize A-COF nanofilm via imine condensation reaction at the gas–liquid interface by the SMAIS strategy using SDBS as the soft-template. As for the B-COF nanofilm, the same method is applied with monomer III (TP) instead of monomer II. To obtain ABA-COF superlattice, layer-by-layer transfer was applied in the order of A-, B-, and A-COF films (Scheme 1b). The same way AAA-, BBB-, and BAB–COF superlattices are also established with the corresponding isolate film.

The successful formation of A- and B-COF films were verified by FT-IR, Raman spectrum. Notably, the FT-IR (or Raman) spectral intensities are weaker than the counterpart COFs powder, indicating the thickness of the COF films is ultra-thin. The band representing C= N stretching vibrational mode is newly formed at 1640 cm^−1^ for A-COF film and the C–N bond is detected for B-COF film in the FT-IR spectra (Fig. S2a) [32, 33]. Meanwhile, the C=N peak at 1606 cm^−1^ and the C–N peak at 1585 cm^−1^ can be also recognized in the Raman spectra of A- and B-COF, respectively (Fig. S2b) [34]. The formation of C=N bond and C–N bond suggesting the success of imine condensation reaction during the synthesis of A- and B-COF films. The XPS analysis was further performed to explore the chemical bonds in A- and B-COF films (Fig. S3). The detection of O in A-COF films is mainly due to the adsorption of oxygen from the air (shown in Fig. S3a). N 1* s* core-level spectra display the peaks at 399.5 eV in A-COF and 399.6 eV in B-COF (shown in Fig. S3b), confirming the C=N and C–N bonds in A- and B-COF skeletons, respectively. Besides, an additional peak of pyridinic-N (398.4 eV) appears in A-COF, confirming the presence of pyridinic-N in A-COF [35]. In addition to the C–N and C=N bonds, C=O bond is detected for B-COF film (Fig. S3c), which is also confirmed by the O 1* s* spectra (Fig. S3d), indicating that the C=O structure from TP is existed in B-COF films. As for the ABA–COF superlattice, all the C–N, C=N, and C=O bonds are detected by Raman spectra (Fig. S2b) and XPS survey spectra (Fig. S3a–d), indicating there have negligible structural damage in the layer-by-layer transfer process.

**Fig. 1:**
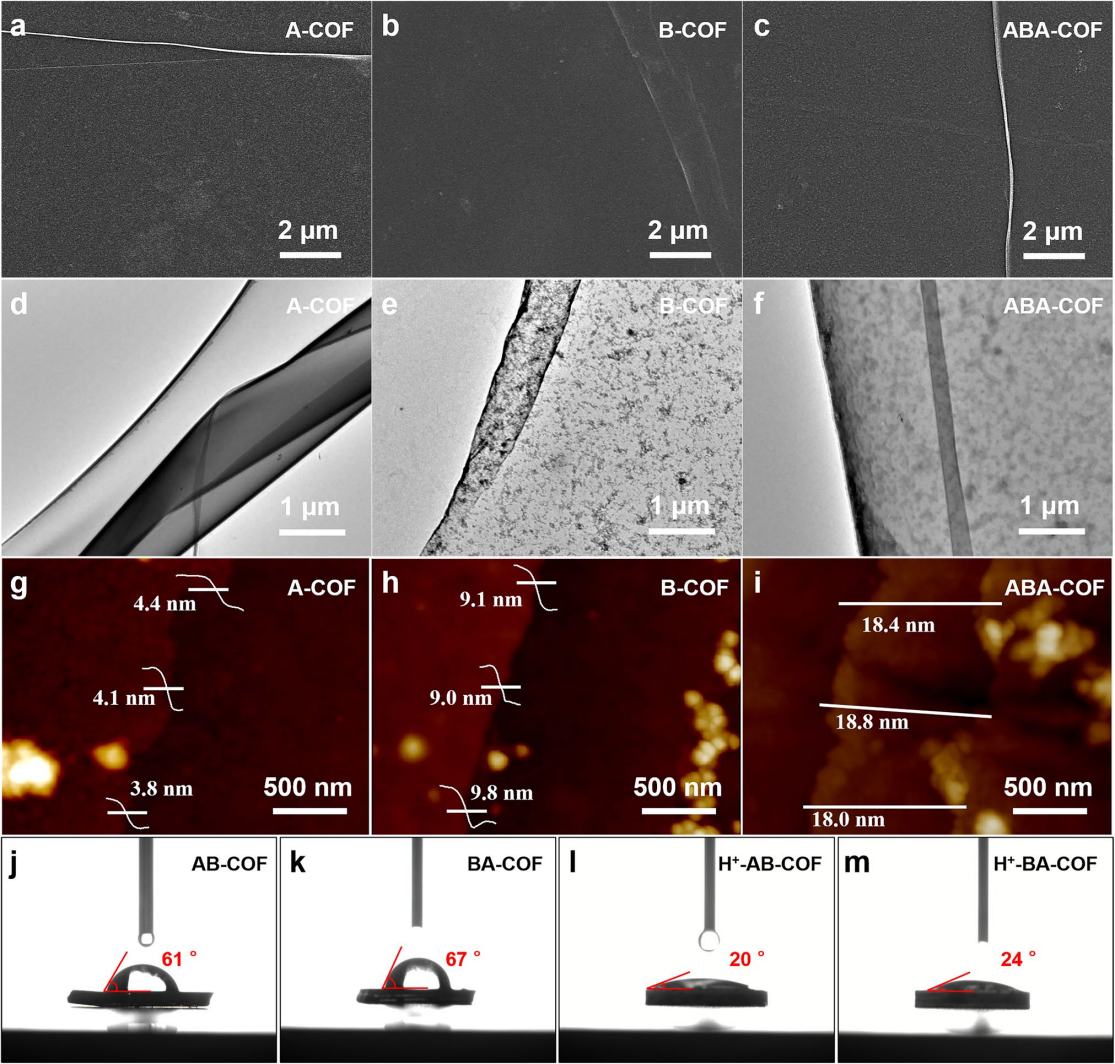
**a–c** SEM, **d–f** TEM and **g–i** AFM images of A-COF film, B-COF film, and ABA-COF superlattice. The contact angle of **j** AB-COF, **k** BA-COF, **l** H^+^-AB-COF and **m** H^+^-BA-COF

To develop bendable MSC with high performance, crack-free superlattice films with large area size and smooth surface are required. The morphologies of synthesized A-COF, B-COF and ABA–COF superlattice films were characterized by OM, SEM, and TEM. As the OM images illustrate (Fig. S4), large pieces of A- and B-COF films are clean and smooth without discernible cracks. The SEM images with high magnification also prove the formation of neat A- and B-COF films (Fig. 1a–b). As for ABA–COF superlattice, the neat surface is remained after the layer-by-layer transfer (Fig. 1c). Detailed morphologies of A-COF, B-COF and ABA–COF superlattice films were characterized by TEM. As shown in Fig. 1d, A-COF film is smooth (Fig. 1d), while slightly rough morphology with black dots is detected for B-COF films (Fig. 1e). The assembled ABA–COF superlattice exhibits large size, uniform morphology, and the structural characteristics of both A- and B-COF films. The blurry dots detected in ABA–COF superlattice suggest that B-COF film are located at the out-up side (Fig. 1f). According to the AFM images shown in Fig. 1g–i, the averaged thickness of A-COF, B-COF and ABA–COF superlattice films are ~ 4.1, ~ 9.3 and ~ 18.4 nm, respectively. The measured thickness of ABA–COF superlattice (~ 18.4 nm) is slightly larger than the sum of corresponding 2A + B COF film layers (17.5 nm), which could be resulted from the *van der Waals* force between layers and surface absorption of water or organic molecules [36, 37].

To simplify the experiment, the wettability tests were conducted on AB- and BA-COF bilayer structures to verify the hydrophilicity of ABA- or BAB–COF superlattices, because of the* Z*-direction symmetry along the interlayer. The contact angle of AB-COF and BA-COF are 61° and 67°, respectively (Fig. 1j–k). AB-COF films are more hydrophilic than BA-COF films, i.e. electrolytes are easier to penetrate into the superlattices with A-COF as the outer layer. After the phosphoric acid treatment, the contact angles are drastically reduced to 20° and 24° (Fig. 1l–m), indicating H^+^ ions in acidic electrolytes can be well immersed into COF superlattice, which is suitable for electrochemical reactions under acidic conditions.

### Electrochemical Performance in a Three-Electrode System

**Fig. 2:**
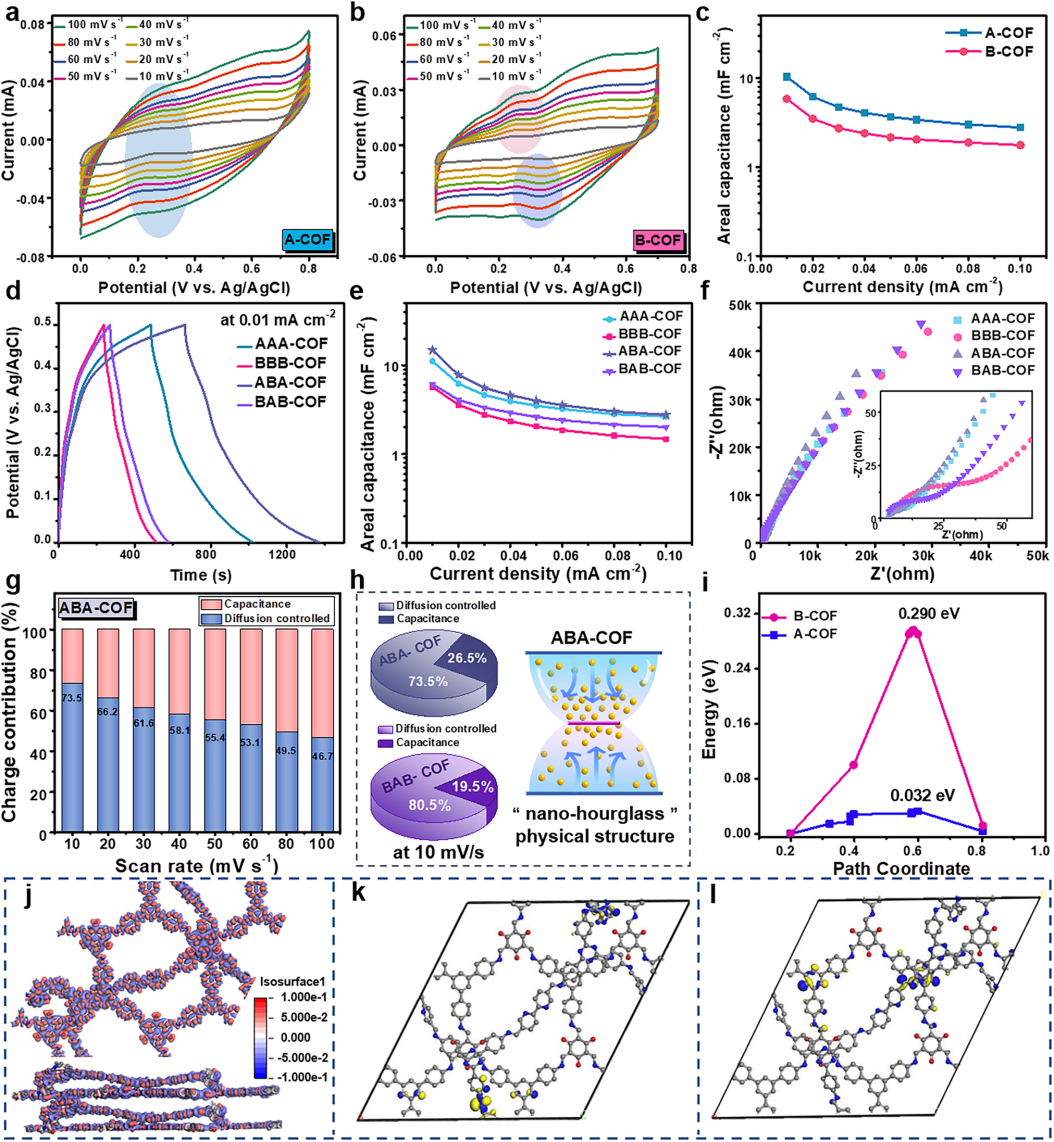
CV curves of **a** A-COF film and **b** B-COF film. **c** *C*_S_ of A- and B-COF films. **d** GCD curves at 0.01 mA cm^−2^, **e** *C*_S_ and **f** Nyquist plots of AAA-, BBB-, ABA- and BAB–COF. superlattices. **g** The diffusion controlled and surface capacitance contribution of ABA–COF superlattice at different scan rates. **h** Pie chart of ABA– and BAB–COF superlattices at 10 mV s^−1^ and an illustration of ABA–COF superlattice internal “nano-hourglass” physical structure. **i** The diffusion barrier of protons, **j** differential charge density distribution (the red region represents charge accumulation, and the blue region indicates charge depletion), and locations of **k** HOMO and **l** LUMO in ABA–COF superlattice

The electrochemical properties of COF superlattices and A- or B-COF films were evaluated by CV, GCD and EIS in a three-electrode system. The A-COF film exhibits the quasi-rectangular CV curves (Fig. 2a), confirming the electrochemical double-layer capacitive property. Moreover, the slight charge transfer peaks appear at the CV potential window of 0.2–0.4 V, which is attributed to the pseudocapacitive of pyridinic-N and the interaction between pyridinic-N and H^+^ [38–40]. And the retention of redox peaks as increasing scan rate indicates that the pyridinic-N structure in A-COF can provide H^+^ diffusion channels and strong affinity. By contrast, a more pronounced pseudocapacitive property is detected for B-COF film due to the detection of stronger redox peaks, which is attributed to abundant active sites for electron transfer and *π*-electron conjugation and the redox reaction caused by the N–H groups and carbonyl groups in the *β*-keto-enamine skeleton (Fig. 2b) [41, 42]. The pseudolinearity of GCD curves can also be used to assess pseudocapacitance of A- and B-COF films (Fig. S5a–b) [39]. The details charge storage ability was further evaluated from GCD curves at various current densities [43]. Based on the discharge time of GCD curves, the *C*_S_ of A-COF film is superior to B-COF at the same current density (Fig. 2c). The electrode–electrolyte interface kinetics of A- and B-COF films were studied by EIS. The Nyquist plots of both films show negligible semicircle at the high-frequency region, indicating the low charge transfer resistance (Fig. S5c), which is attributed to fast charge transfer at the electrode/electrolyte interfaces due to the *π* electronic conjugation along the extended porous network of A- and B-COF [43, 44].

The assembled AAA-, BBB-, ABA-, and BAB–COF superlattices were characterized by the identical electrochemical tests. The CV curves of the AAA- and BBB–COF superlattices exhibit pronounced characteristics of the A- and B-COF films with the increase of corresponding COF film layers, respectively (Fig. S6a–b). For ABA–COF superlattice, the CV curves similar to that of AAA–COF indicates that the electrochemical performance of the outer layer A-COF is dominant, which may mask the redox peak of B-COF (Fig. S6c). Likewise, the CV curves of BAB–COF superlattice exhibit substantial redox peaks in accord with BBB–COF superlattice (Fig. S6d). These results indicate that the three-layers superlattice system mainly exhibits the capacitive behavior of outermost COF layers.

In addition, the slight deformation and pseudolinearity shape are obtained for the GCD curves of these four superlattices at different current densities (Fig. S6e–h), confirming the existence of faradaic redox behavior in all the superlattices. Taking the GCD curve at 0.01 mA cm^−2^ for four superlattices (Fig. 2d), the ABA–COF has the longest discharge time, corresponding to the highest *C*_S_ values at the same current density (Fig. 2e). The differences in *C*_S_ values can be explained by the unique structure and redox functional groups in the superlattices. As for the ABA–COF structure, (i) the outer A-COF film and the inner B-COF film provide fast charge transfer channels with pronounced electrochemical double-layer capacitance and abundant charge transfer redox reactions with pronounced pseudocapacitance, respectively. And the A-COF structure is more stable during the reaction process than that of the B-COF, which ensures the superlattice structure stability with A-COF as the outermost layer during the charge/discharge reaction; (ii) the outer A-COF owns the mesoporous and regular channels, contributing to substantial electrolyte H^+^ ions to shuttle into the pore channels and rapid transfer to the internal B-COF interface [45], and the restriction of B-COF micropores allows the interior structure to obtain high ion concentration. The nanoscale porous channels are guaranteed to shorten the diffusion distance of protons to the active center and achieve charge accumulation, accelerating the redox reaction rate to realize efficient energy storage [46]. When the B-COF film is located in the outermost layer, the dense microporous channels will restrict the rapid entry of H^+^ cations into the superlattice interior and obtain limited charge storage, which can be verified by the lower areal capacitance of BAB- and BBB–COF superlattice. In addition, the BAB–COF superlattice shows higher capacitance than BBB–COF, confirming that the synergistic effect of varied pore size and redox active groups in the superlattices assembled by different COF films can bring higher charge storage capacity.

Furthermore, the Nyquist plots of all these superlattices show that the ABA–COF superlattice possesses the lowest curvature diameter at the high-frequency region, indicating the lowest charge transfer resistance (Fig. 2f). Compared with other superlattices, the ABA–COF superlattice exhibits the highest slope at the low-frequency region, confirming the low *Warburg* impedance related to the rapid electrolyte ion diffusion at the electrode interface [44]. These results including CV, GCD, and EIS confirm that the electrochemical performance of the ABA–COF superlattice is superior to other three AAA-, BBB-, and BAB–COF superlattices.

### Investigation of the Charge Storage Mechanism

The surface and diffusion-controlled capacitance contributions were calculated based on the CV curve, for further quantitative verify the charge storage mechanism. In Fig. 2g, the diffusion-controlled contribution accounts for 73.5% at 10 mV s^−1^, and decreases to 46.7% with the scan rate increasing to 100 mV s^−1^, revealing the diffusion-controlled dominant energy storage mechanism for the ABA–COF superlattice. The charge storage contributions of the other AAA-, BBB- and BAB–COF superlattices at different scan rates also exhibit the dominant trend of diffusion-controlled shown in Fig. S7a–c. The charge storage contributions of representative ABA- and BAB–COF superlattices were compared at the scan rate of 10 mV s^−1^. The ABA–COF superlattice has a 7.0% higher percentage of surface capacitance than BAB–COF, which can be mainly attributed to the increased contribution of pseudocapacitance in this nanoscale structure [47], confirming the microporous charge accumulation effect of mesoporous–microporous stacking in its internal “nano-hourglass” physical structure (Fig. 2h) [26].

To further reveal the proton transfer behavior between the A- and B-COF channels, the diffusion barrier was stimulated by DFT calculation. There are two proton diffusion pathways between the ABA–COF superlattice (protons pass through adjacent sites in the intine of A- or B-COF channel along vertical paths) (shown in Fig. S8). As shown in Fig. 2i, the proton diffusion barrier along A-COF (0.032 eV) is much lower than that of B-COF (0.290 eV). The low barrier indicates that protons can conduct rapidly in the vertical channel [12]. In addition, as shown in the differential charge density distribution (Fig. 2j), there is charge accumulation and consumption at the junction of A- and B-COF (the red region represents charge accumulation, and the blue region indicates charge depletion), which can also be confirmed by the locations of the HOMO and LUMO energy levels in the ABA–COF superlattice (Fig. 2k–l). The HOMO level is mainly distributed on the bipyridine skeleton, while the LUMO level is derived from B-COF, which indicates that A-COF is the electron donor and transfers the charge between A- and B-COF.

Combining the above experimental results and simulation calculations, the fast charge transfer and preeminent energy storage ability is result from: (i) protons in the superlattice can be rapidly transferred to the B-COF along the intine and pore of channels of the outside A-COF. (ii) the offset of potential barriers leads to the accumulation of protons on the B-COF surface, and redox reactions can be fully carried out, resulting in higher capacitance [48]. In other words, in this “nano-hourglass” geometrical configuration, the A-COF layer can locally trap the electrolyte as an energy storage layer and limit the charge relief, and promote electrolyte transferring to the B-COF layer, realizing the charge accumulation and exhaust it at the active site during the redox reaction. Moreover, in the case that the micropore cannot fully withstand the highly loaded electrolyte from the mesopore, the micropore B-COF is protected into the sandwich of the two outer mesopores A-COF [49], thus avoiding the strain of internal B-COF and energy storage degradation during repeated charge/discharge cycles [45], further ensuring efficient energy storage in ABA–COF superlattice.

### Flexibility and Application of the Fabricated MSC Device

**Fig. 3:**
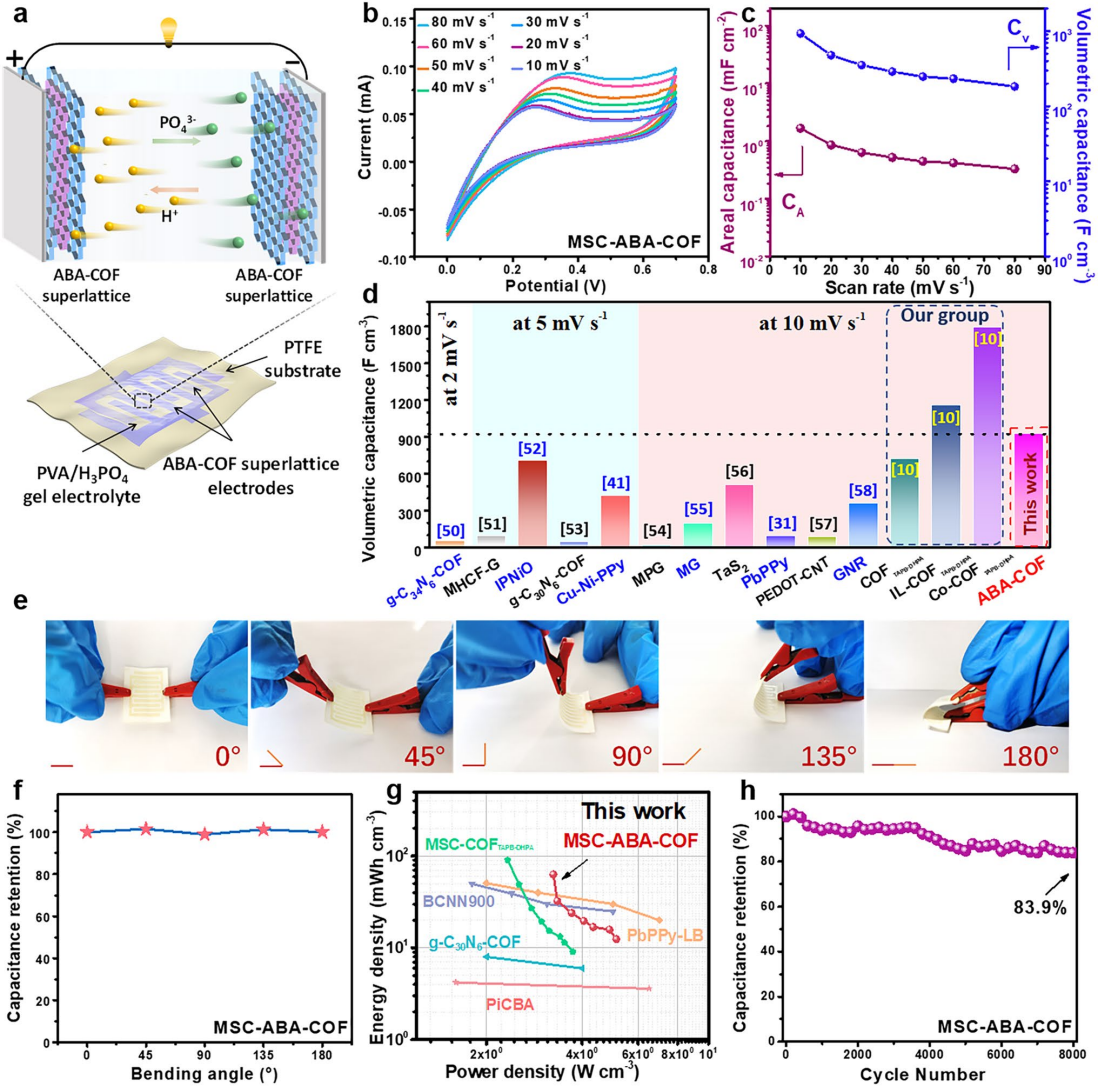
**a** Schematic diagram of the in-plane MSC configuration. **b** CV curves, **c** *C*_A_ and *C*_V_ of MSC–ABA–COF. **d** *C*_V_ comparison with some reported MSC devices. **e** Optical photographs and **f** capacitance retention under different bending degrees of MSC–ABA–COF. **g** *Ragone* plots of MSC–ABA–COF, as well as some reported MSC devices. **h** Cycling stability of MSC–ABA–COF

The optimized ABA–COF superlattice was applied as interdigital electrodes to assemble the corresponding flexible MSC device (defined as MSC–ABA–COF), using PTFE membrane as substrate and gel H_3_PO_4_/PVA as electrolyte (shown schematically in Fig. 3a). The electrochemical performance was evaluated by the CV technique at given scan rates of 10 to 80 mV s^−1^. The CV curves show significant redox peaks, emphasizing the faradaic redox reaction properties. In addition, the almost invariant CV shape confirms the good capability of this MSC–ABA–COF device (Fig. 3b). According to the CV curves, the *C*_A_ and the corresponding *C*_V_ values at different scan rates of MSC–AB–COF were also evaluated to avoid thickness differences. As shown in Fig. 3c, the MSC–ABA–COF exhibits a comparable *C*_A_ of 1.7 mF cm^−2^ at 10 mV s^−1^, and the highest *C*v of 927.9 F cm^−3^ at 10 mV s^−1^ than reported two-dimensional alloy, graphite-like carbon and undoped COF-based MSC devices so far (Fig. 3d) [31, 41, 50–58]. The unique superlattice structure generates excellent charge storage capacity, resulting in the high capacitance of MSC–ABA–COF [59].

Lightweight flexible properties and good bending resistance are essential for MSCs in practical applications [60]. To meet the demand for future miniaturization and wearable electronics applications, the capacitance retention of MSC–ABA–COF was also evaluated by CV technique at 80 mV s^−1^ under different bending angles of 0°–180°, as shown in Fig. 3e–f. It can be observed that there is no significant difference in capacitance values, indicating that the ABA–COF superlattice is not damaged even after high-angle and repeat arbitrary bending (Fig. S9). The MSC–ABA–COF can maintain its performance under different bending degrees, displaying excellent mechanical flexibility and outstanding stability, indicating its potential for application in flexible electronic devices [61].

Meanwhile, Fig. 3g collects the *Ragone* plots with two important parameters of the energy and power density for MSC–ABA–COF and compared with reported MSC devices. The MSC–ABA–COF delivers the energy density of 63.2 mWh cm^−3^ at 3.3 W cm^−3^ power density. At a certain power density range, the MSC–ABA–COF exhibits a higher competitive energy density than most MSCs with higher energy density reported so far, such as PbPPy-LB [31], BCNN900 [62], g-C_30_N_6_-COF [53], PiCBA [63]. In addition, the long-term cycle stability of MSC–ABA–COF was also evaluated by the capacitance retention after CV charge/discharge cycles. The capacitance retention was about 83.9% of the initial capacitance after 8,000 charge/discharge cycles at 80 mV s^−1^ (Fig. 3h). Considering the lack of reported similar MSCs-based self-supported COF film electrodes, the capacitance retention of MSC–ABA–COF was compared with our recently reported MSCs assembled by the single COF film, such as MSC–COF_TAPB-DHPA_ (97% after 5000 cycles) and MSC–Co-COF_TAPB-DHPA_ (80% after 5000 cycles). The results show that the MSC–ABA–COF owns good stability under longer-term operations, even if there are multiple laminated COF layers.

## Conclusions

In conclusion, two free-standing films mesoporous A-COF with imine bond linkage and microporous B-COF with *β*-keto-enamine-linkages were synthesized by the SMAIS method. Based on two capacitance contribution mechanisms, the A- and B-COF films were assembled into ABA–COF superlattice for the first time by layer-by-layer transfer. The electrochemical characterization of superlattices assembled with mesoporous–microporous “nano-hourglass” geometric construction verified that the geometry-induced rapid charge transfer/accumulation can improve the synergistic effect of double layer capacitance and faradic redox reaction. The resulting in-plane flexible MSC–ABA–COF delivered a 927.9 F cm^−3^ specific volumetric capacitance, a high energy density of 63.2 mWh cm^−3^ and excellent bending resistance performance. This work provides a potential direction for 2D COF-based superlattices self-supported electrodes in wearable MSC devices. Moreover, this superlattice preparation strategy provides a new way for the application of functionalized COF films in the fields of energy storage/conversion and catalysis.

### Supplementary Information

Below is the link to the electronic supplementary material.

Supplementary file1 (PDF 1028 kb)
