# Supplementary material for: Bending Resistance Covalent Organic Framework Superlattice: “Nano-Hourglass”-Induced Charge Accumulation for Flexible In-Plane Micro-Supercapacitors
Source: Nanomicro Lett. 2022 Dec 30;15:25. doi: 10.1007/s40820-022-00997-0 (PMC9803805; doi:10.1007/s40820-022-00997-0)
Supplement: Supplementary file 1 — Supplementary file1 (PDF 1028 kb) [file 40820_2022_997_MOESM1_ESM.pdf]

Supporting Information for

Bending Resistance Covalent Organic Framework Superlattice: "Nano-Hourglass"-Induced Charge Accumulation for Flexible In-Plane Micro-Supercapacitors

Xiaoyang Xu[1, 3, #], Zhenni Zhang[1, #], Rui Xiong[1, #], Guandan Lu[1], Jia Zhang[1], Wang Ning[1, 3], Shuozhen Hu[2, *], Qingliang Feng[3, *], Shanlin Qiao[1, 4, *]

[1] College of Chemistry and Pharmaceutical Engineering, Hebei University of Science and Technology, Shijiazhuang, 050018, P. R. China

[2] State Key Laboratory of Chemical Engineering, East China University of Science and Technology, Shanghai 200237, P. R. China

[3] School of Chemistry and Chemical Engineering, Northwestern Polytechnical University

Xi'an 710072, P. R. China

[4] Hebei Electronic Organic Chemicals Engineering Center, Shijiazhuang 050018, P. R. China

[#] Xiaoyang Xu, Zhenni Zhang, and Rui Xiong contributed equally to this work.

* Corresponding authors. E-mail: ccpeslqiao@hebust.edu.cn (S. Q.), fengql@nwpu.edu.cn (Q. F.), shuozhen.hu@ecust.edu.cn (S. H.)

Supplementary Figures and Table

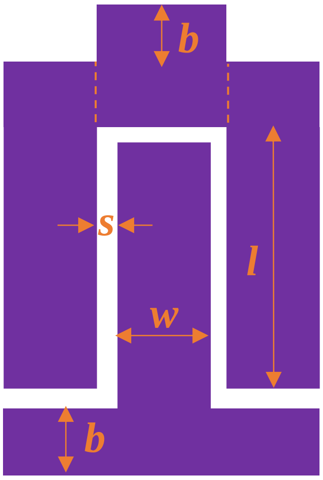

Interdigital Finger Numbers, N	10
Finger Width, *w* /mm	2
Finger Length, *l* /mm	15
Interspatial Distance, *s* /mm	0.6
Edge Breath, *b* /mm	1.5
Effective Area /cm^2	3.78

Fig. S1 The parameters of the prepared interdigitated electrodes with a custom-made interdigital mask

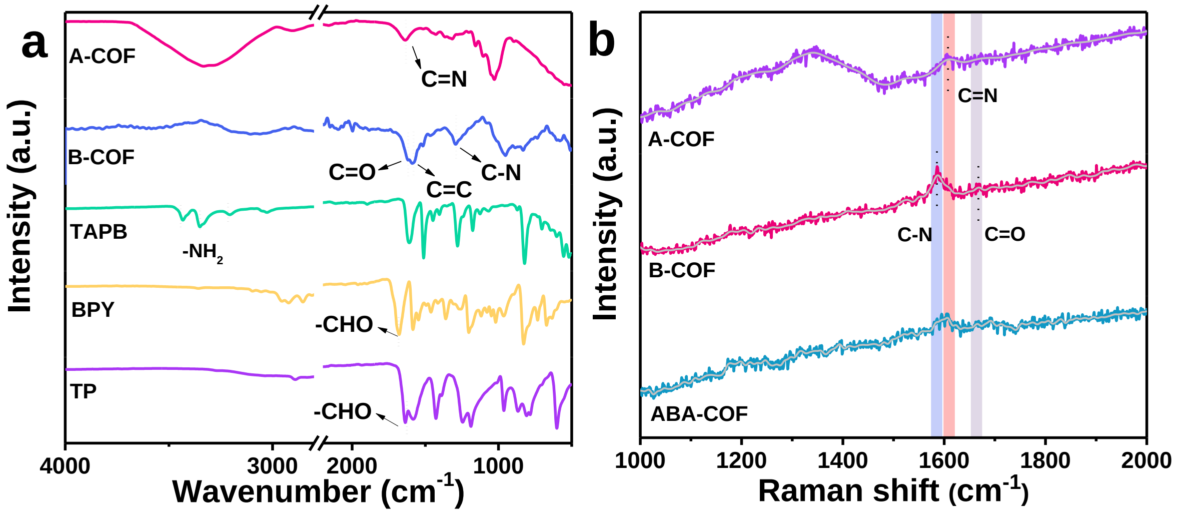

Fig. S2 FT-IR spectra (**a**) of A- and B-COF films. Raman spectra (**b**) of A-COF, B-COF and ABA-COF superlattice films

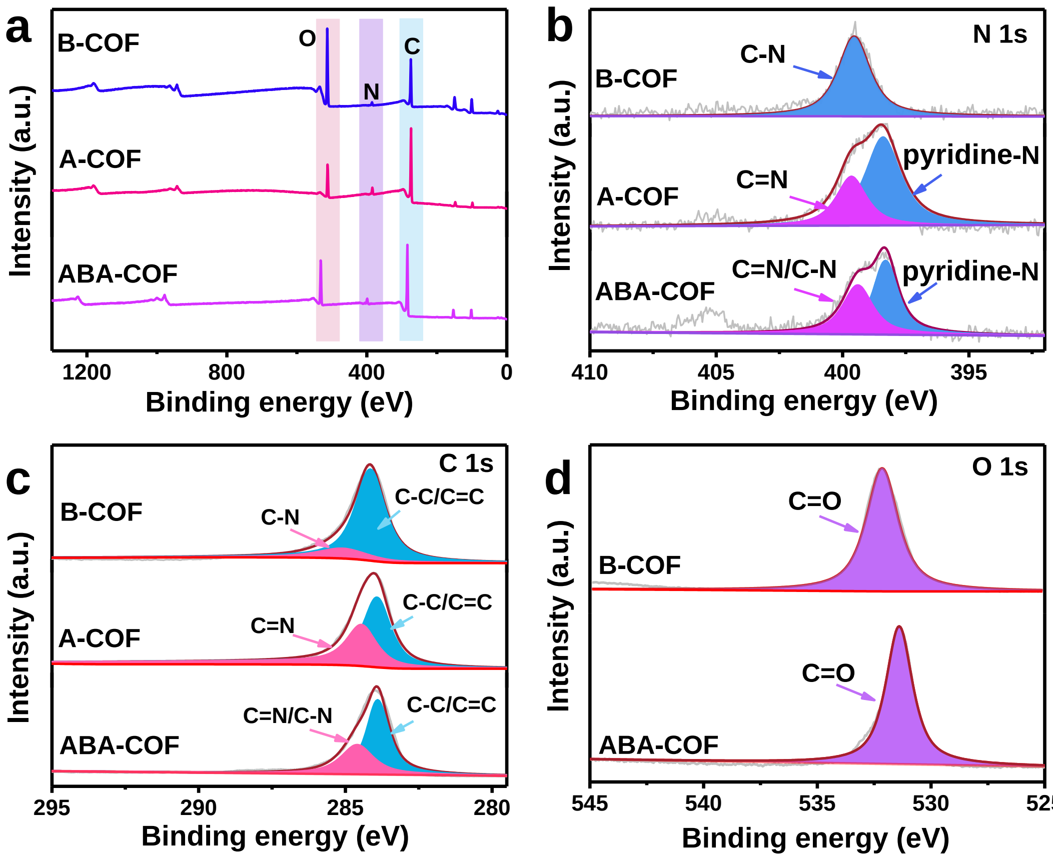

Fig. S3 XPS survey spectra (**a**), N 1s (**b**), and C 1s (**c**) of of A-COF, B-COF and ABA-COF superlattice films; O 1s (**d**) of B-COF and ABA-COF superlattice films

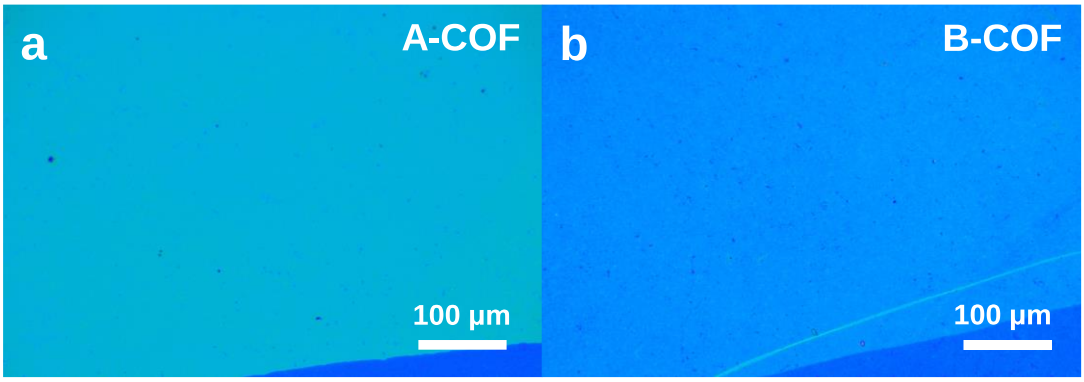

Fig. S4 OM images of A-COF (**a**) and B-COF (**b**) films

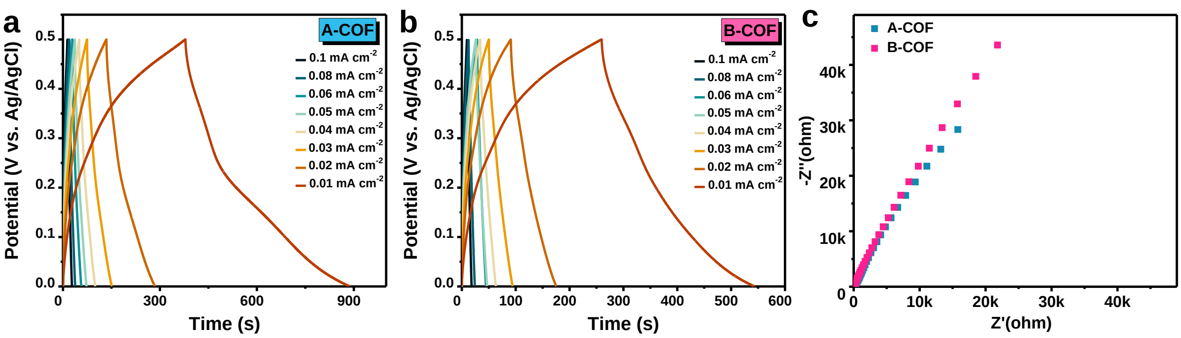

Fig. S5 GCD curves (**a-b**) and Nyquist plots (**c**) of A- and B-COF films

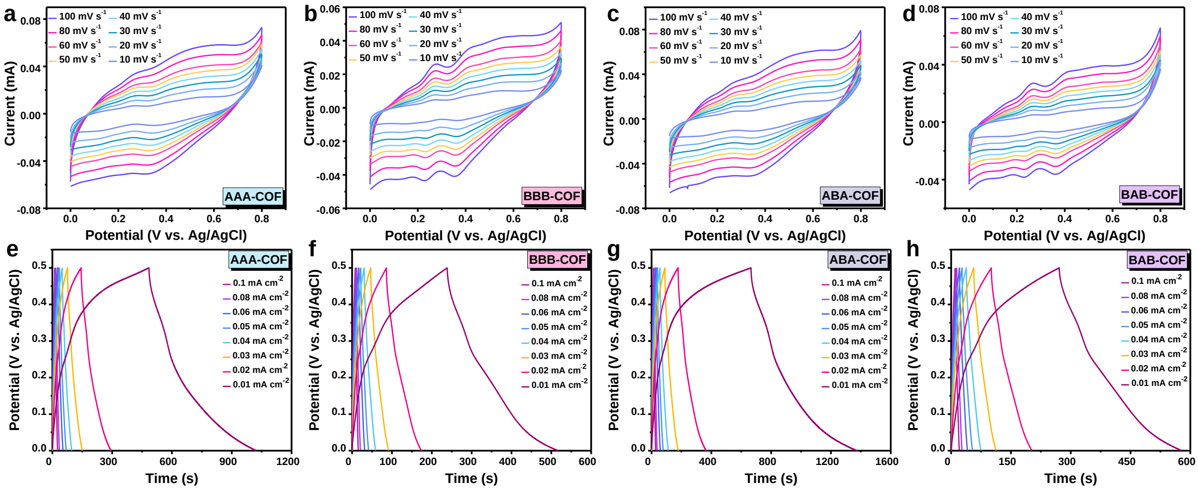

Fig. S6 CV (**a-d**) and GCD (**e-h**) of AAA-, BBB-, ABA- and BAB-COF superlattices

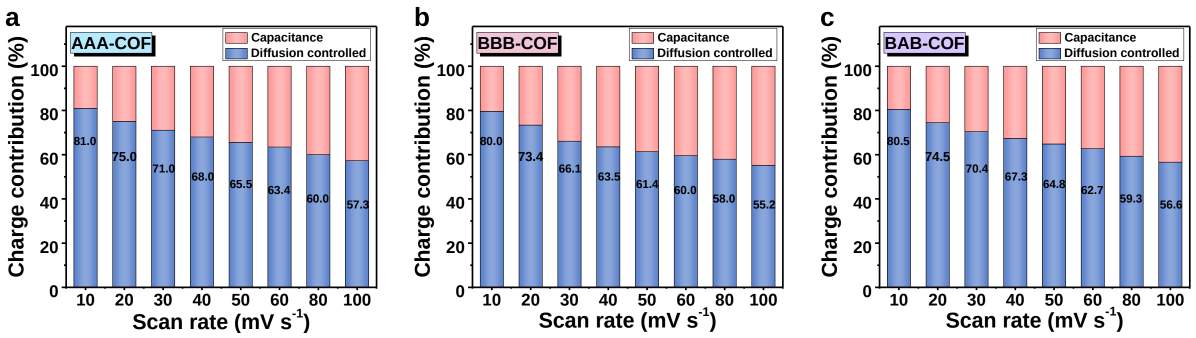

Fig. S7 The diffusion-controlled and surface capacitance contribution of AAA-COF (**a**), BBB-COF (**b**) and BAB-COF (**c**) superlattices

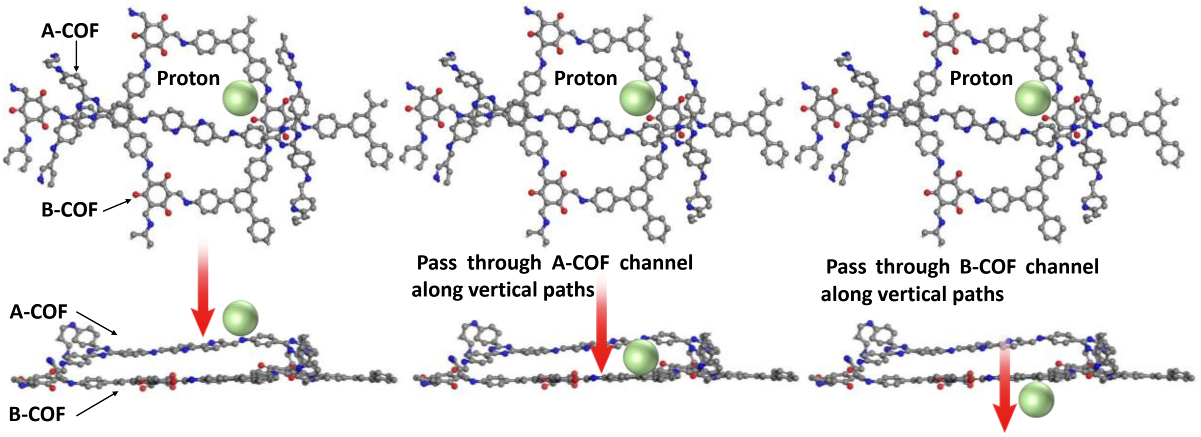

Fig. S8 The diagram of that proton passes through adjacent sites in the intine of A-COF and B-COF channel along vertical paths.

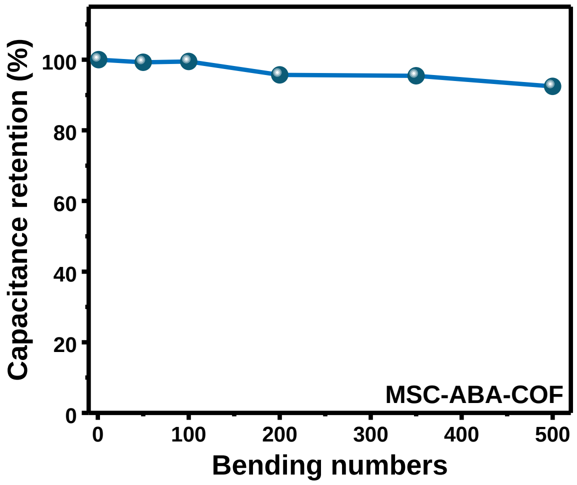

Fig. S9 Capacitance retention of MSC-ABA-COF after repeat bending

Table S1 The performance of recent reported 2D materials based MSCs and this work. ([(a)] mV s^{-1}; [(b)] mF cm^{-2}; [(c)] F cm^{-3}; [(d)] mWh cm^{-3}; [(e)] W cm^{-3})

Electrode	Electrolyte	Scan rate [(a)]	C_A [(b)]	C_V [(c)]	E_V [(d)] (P_V [(e)])	Thickness	Capacitance retain	References
MPG	PVA/H_2SO_4	10	0.08	17.9	2.5 (495)	15 nm	98.3% after 100,000 cycles (50 V s^{-1})	Nat. Commun. 2013, 4, 2487.
PiCBA	PVA/H_2SO_4	50	0.171	34.1	4.7 (1323)	50.0 nm	86% after 350 cycles (50 mV s^{-1})	Angew. Chem. Int. Ed. 2017, 56 (14), 3920-3924.
EG/MXene	PVA/H_3PO_4	0.2 A/cm^3	N_A	184	3.4 (1.6)	2.5 μm	85.2% after 2,500 cycles (10 mV s^{-1})	Adv. Energy Mater. 2017, 7(4), 1601847.
MG-MSC	Ionogel electrolyte	10	0.338 (PVA/H_2SO_4)	197 (PVA/H_2SO_4)	23 (1860)	N_A	96% after 20,000 cycles (100 V s^{-1})	Adv. Mater. 2018, 30(27), 1801384.
TaS_2	PVA/LiCl	10	N_A	508	58.5 (1.316)	330 nm	92% after 4,000 cycles (10 mV s^{-1})	J. Am. Chem. Soc. 2018, 140(1), 493-498.
g-$C_{34}N_6$-COF	PVA/LiCl	2	15.2	50.7	7.3 (0.05)	3 μm	93.1% after 5,000 cycles (10 mV s^{-1})	Angew. Chem. 2019, 58 (35), 12065-12069.
PEDOT-CNT	PVA/H_2SO_4	10	20.6	82.4	11.4 (18.55)	2.5 μm	99.9% after 20,000 cycles (10 mV s^{-1})	Nanoscale 2019, 11 (16), 7761-7770.
IPNiO	SMPG–EGS	5	155	705	60 (70)	44.8 nm	≈90% after 8,000 cycles (10 mV s^{-1})	J. Mater. Chem. A 2019, 7 (37), 21496-21506.
g-$C_{30}N_6$-COF	$EMIMBF_4$/PVDF-HFP	5	44.3	44.3	38.5 (0.3)	10 μm	95% after 5,000 cycles (10 mV s^{-1})	Sci. Bull. 2020, 65 (19), 1659-1666.
PbPPy	$EMIMBF_4$	10	0.95	91.4	50.7 (1.83)	104 nm	85% after 10,000 cycles (10 mV s^{-1})	Adv. Funct. Mater. 2019, 30 (7), 1908243.
α-Co$(OH)_2$/RGO	PVA/KOH	0.5 mA cm^{-2}	130	260	20 (1.12)	11.6~13.5 nm	99.35% after 2,000 cycles (1 mA cm^{-2})	J. Colloid Interface Sci. 2021, 598, 1-13.
BCNN900	$EMIMBF_4$/PVDF-HFP	0.25 mA cm^{-2}	80.1	133.5	67.6 (0.8)	~6 μm	~91% after 10,000 cycles (10 mV s^{-1})	Energy Storage Mater. 2021, 42, 430-437.
rGO/GO film	(PVA)/H_2SO_4	0.25 mA cm^{-2}	94.8	NA	10.7 (0.113)	120~200 mm	57.1% after 20,000 cycles (10 mV s^{-1})	Carbon 2021, 175, 27-35.

SPG-MSC	PVA/H_3PO_4	5	1.0	2	1.81 (297)	5 µm	91.8% after 10,000 cycles (0.2 mA cm^{-2})	Energy Environ. Sci. 2019, 12(5), 1534-1541.
MHCF/graphene	PVA/LiCl	5	19.8	93.2	44.6 (0.34)	~2 µm	96.8% after 5,000 cycles (10 mV s^{-1})	Mater. Horiz. 2019, 6(5), 1041-1049.
GEG	KTFSI-P14-TFSI	10	-	13.7	14.5 (5.19)	3 nm	93% after 10,000 cycles (10 µA cm^{-2})	Carbon 2022, 196, 203-212.
Cu-Ni-PPy	$LiClO_4$/PVA	5	126.67	422	31.78 (0.15)	3 µm	89.3% after 10,000 cycles (50 mV s^{-1})	Energy Storage Mater. 2022, 51, 139-148.
GNRs	H_2SO_4/PVA	10	3.6	355	8 (550)	100 nm	93% after 10,000 cycles (1 V s^{-1})	Adv. Funct. Mater. 2022, 32 (16) 2109543
COE-4	H_2SO_4/PVA	0.04 mA cm^{-2}	14.3	15.7	1.02 (2.37)	9.1±1.7 µm	87.3% after 5,000 cycles (50 mV s^{-1})	Energy Technol. 2022, 10 (5), 2200133
$COF_{TAPB-DHPA}$	PVA/H_3PO_4	10	0.8	723.2	90.7 (2.3)	11.0 nm	97% after 5,000 cycles (1 V s^{-1})	Chem. Eng. J. 2022, 447, 137447.
IL- $COF_{TAPB-DHPA}$			1.4	1157.9	139.7 (3.6)	11.7 nm	80% after 5,000 cycles (1 V s^{-1})	
Co-$COF_{TAPB-DHPA}$			1.8	1790.1	230.4 (5.9)	10.0 nm	80% after 5,000 cycles (1 V s^{-1})	
**ABA-COF superlattice**	**PVA/H_3PO_4**	**10**	**1.7**	**927.9**	**63.2 (3.3)**	**18.4 nm**	**83.9% after 8,000 cycles (80 mV s^{-1})**	**This work**
